# Development and Validation of a Digital (Peek) Near Visual Acuity Test for Clinical Practice, Community-Based Survey, and Research

**DOI:** 10.1167/tvst.11.12.18

**Published:** 2022-12-30

**Authors:** Marzieh Katibeh, Sandip Das Sanyam, Elanor Watts, Nigel M. Bolster, Reena Yadav, Abhishek Roshan, Sailesh K. Mishra, Matthew J. Burton, Andrew Bastawrous

**Affiliations:** 1 Peek Vision, Berkhamsted, UK; 2 Department of Ophthalmology, Aarhus University Hospital, Aarhus, Denmark; 3 Sagarmatha Choudhary Eye Hospital, Lahan, Nepal; 4 Tennent Institute of Ophthalmology, Glasgow, UK; 5 International Centre for Eye Health, Clinical Research Department, London School of Hygiene and Tropical Medicine, London, UK; 6 Nepal Netra Jyoti Sangh, Kathmandu, Nepal; 7 National Institute for Health Research Biomedical Research Centre for Ophthalmology at Moorfields Eye Hospital NHS Foundation Trust and UCL Institute of Ophthalmology, London, UK

**Keywords:** vision, visual acuity, near vision, screening, test, mHealth, digital

## Abstract

**Purpose:**

Unaddressed near vision impairment (NVI) affects more than 500 million people. Testing near vision is necessary to identify those in need of services. To make such testing readily accessible, we have developed and validated a new smartphone-based near visual acuity (NVA) test: Peek Near Vision (PeekNV).

**Methods:**

Two forms of the PeekNV test were developed: (1) quantitative measurement of NVA, and (2) binary screening test for presence or absence of NVI. The validity study was carried out with 483 participants in Sagarmatha Choudhary Eye Hospital, Lahan, Nepal, using a conventional Tumbling “E” Near Point Vision Chart as the reference standard. Bland–Altman limits of agreement (LoA) were used to evaluate test agreement and test–retest repeatability. NVI screening was assessed using Cohen's kappa coefficient, sensitivity, and specificity.

**Results:**

The mean difference between PeekNV and chart NVA results was 0.008 logMAR units (95% confidence interval [CI], −0.005 to 0.021) in right eye data, and the 95% LoA between PeekNV and chart testing were within 0.235 and −0.218 logMAR. As a NVI screening tool, the overall agreement between tests was 92.9% (κ = 0.85). The positive predictive value of PeekNV was 93.2% (95% CI, 89.6% to 96.9%), and the negative predictive value 92.7% (95% CI, 88.9% to 96.4%). PeekNV had a faster NVI screening time (11.6 seconds; 95% CI, 10.5 to 12.6) than the chart (14.9 seconds; 95% CI, 13.5 to 16.2; *P* < 0.001).

**Conclusions:**

The PeekNV smartphone-based test produces rapid NVA test results, comparable to those of an accepted NV test.

**Translational Relevance:**

PeekNV is a validated, reliable option for NV testing for use with smartphones or digital devices.

## Introduction

The World Health Organization (WHO), in the *International Classification of Diseases, 11th Revision* (ICD-11), defined near vision impairment (NVI) as “presenting near visual acuity worse than N6” at 40 cm, elsewhere described as “presenting near visual acuity worse than N6 or 0.8M with existing correction.”^1^^,^^2^ This equates to a logMAR of approximately 0.27 (Table 1). Although distance vision is more often assessed, NVI has the potential to substantially limit function and quality of life, as well as population-level productivity.^3^^,^^4^ For example, trial evidence shows that the provision of presbyopia correction can increase productivity for agricultural activities requiring good near vision.^5^ Presbyopia poses a barrier to a diverse range of activities, including reading, writing, use of mobile phones, interpretation of facial expressions, and close practical tasks such as sewing. In addition to practical and social difficulties, NVI can be dangerous—for example, with an increased risk of accidental ingestion of foreign bodies.^6^

**Table 1.** Conversion of NVA Measurement Units

N Units	M Units	LogMAR at 40-cm Viewing Distance (to Two Decimal Places)
N64	8M	1.30
N50.4	6.3M	1.20
N40	5M	1.10
N32	4M	1.00
N25.6	3.2M	0.90
N20	2.5M	0.80
N16	2M	0.70
N12.8	1.6M	0.60
N10	1.25M	0.50
N8	1M	0.40
**N6**	**0** **.75M**	**0.27**
N3.2	0.40M	0.00

N units are typeface points for lowercase letters; M units are the distance in meters at which the outer diameter subtends 5 minutes of arc. N-score = tan (antilog(logMAR score)/60) × 400 mm × 5/0.18175. Bold terms indicate the optotype size to be tested when assessing for NVI.

Uncorrected presbyopia was estimated to be responsible for 510 million of the world's 1.1 billion people with visual impairment in 2020.^7^ In most, presbyopia is easily addressed by the provision of near correction spectacles. The unmet need for optical correction varies substantially among regions, as it is higher in low- and middle-income countries and approaches 90% in central Sub-Saharan Africa.^7^^,^^8^ Following the release of the World Report on Vision in 2019, NVI has been prioritized by the WHO, with major emphasis being placed on the global reporting of effective refractive error coverage (eREC).^9^ WHO strongly urges countries to measure and report both near and distance eREC at the population level.^10^ Therefore, there is a need for reliable tools for community vision screening and population-based surveys, such as the Rapid Assessment of Avoidable Blindness (RAAB), to identify people with NVI and enable estimation of eREC for distance and near.^11^ The ideal tool for this task should provide a quick and reliable test of distance visual acuity and near visual acuity (NVA) that can be easily integrated into testing methodology, including increasingly favored digital data collection tools, and which meets the Visual Acuity Measurement Standard, as set out by the International Council of Ophthalmology.^12^

Despite the impact of presbyopia on the lives of so many people, testing of NVA is much less standardized than that for distance vision. Most existing chart and digital NVA tests are found to have minimal or no evidence of validation.^13^^,^^14^ In addition to NVA tests, near reading acuity test charts are available; however, these are language specific and dependent on literacy, and they test cognitive functions beyond vision. Smartphones and tablets can also be used to test vision, allowing for automated digital data collection and transfer. Although smartphone access at one point posed a barrier to use in low- and middle-income countries, two-thirds of the global population now own a mobile phone, with a predicted 39% of the population accessing mobile internet by 2025 in Sub-Saharan Africa, as well as the uptake of a WHO smartphone hearing test in 179 of 195 countries globally.^15^^–^^17^ The ability to clearly see phone and computer screens is now a priority for many people. Technology has proven useful in testing distance vision in a wide range of environments and contexts, including community and school eye health programs and population surveys, as has been successfully demonstrated by tests including Peek Acuity and Peek Contrast Sensitivity.^18^^–^^22^ Of tests of its type, Peek Acuity has been shown to have superior test–retest reliability and the strongest correlation with Early Treatment of Diabetic Retinopathy Study (ETDRS) visual acuities measured in clinics, according to a recent systematic review.^23^ The aim of this study was to design and validate an equivalent digital tool for measuring near vision, with attributes comparable to those of an accepted conventional NVA testing method.

## Methods

### Product Development

Following literature review, international experts in the field of eye health programs and digital technology were surveyed for their views regarding the characteristics of an ideal NVA test. Survey respondents included internal experts within Peek (including optometrists and ophthalmologists working internationally, researchers, and software developers) and external experts working at the London School of Hygiene and Tropical Medicine International Centre for Eye Health, the International Myopia Institute, Brien Holden Vision Institute, and WHO. Some respondents were interviewed further. Peek Near Vision (PeekNV) was developed by combining the results from the above with existing Peek Acuity technology. Based on the review and expert opinion, the following desirable test characteristics were identified:

• Provides full quantitative NVA measurement
• Identifies NVI at a threshold for onward referral for services
• Prompts provision of prescription-ready readers
• Can be integrated into population survey data collection to measure the frequency of NVI
• Uses Tumbling “E” single optotype test as the preferred format
• Usable in populations with low levels of literacy
• Short test time

### Test Design

A single tumbling “E” optotype is shown in one of four orientations (90°, 180°, 270°, or 360°) to reduce barriers posed by language, literacy, or age. A bounding box simulates the crowding effect of a standard ETDRS chart using a crowding bar, with thickness equivalent to the limb of the optotype, and spacing between optotype and crowding bar equal to that of half the total optotype size. This contour interaction format matches that used by the reference standard chart. Optotypes are presented at the following sizes: N64, N50.4, N40, N32, N25.6, N20, N16, N12.8, N10, N8, N6, and N3.2, following a logarithmic pattern to enable comparison with existing charts and clinical relevance while excluding sizes that could not be accurately portrayed by current smartphone technology due to pixel size.

### Validity Study Design

Although no single gold standard test exists for NVA, a conventional standard test was selected that could fulfill these requirements as a comparator: Tumbling “E” Near Point Vision Chart (Precision Vision, Woodstock, IL). Two sub-studies separately assessed (1) the use of PeekNV for near vision screening (a binary screening test for presence or absence of NVI), and (2) as a quantitative measurement of NVA. Sample size was calculated for sub-study 1 using kappa statistics as per Fleiss’ formula and for sub-study 2 based on Bland–Altman limits of agreement (LoA).^24^^,^^25^ Formulae are available in the Supplementary Materials. These calculations produced minimum required sample sizes of 115 and 273, respectively.

### Participants

This was a cross-sectional observational validity/methods comparison study. Participants were recruited from Sagarmatha Choudhary Eye Hospital outpatient clinic in Lahan, Nepal. Patients with a wide range of eye problems, including refractive error, self-present or are referred to the hospital. No patients who would not otherwise have been attending the clinic were recruited. Other diagnoses and the results of an ophthalmic examination by an ophthalmologist were collected and considered as part of the eligibility assessment. A dedicated coordinator assessed the inclusion and exclusion criteria and obtained informed consent. The inclusion and exclusion criteria were as follows.

#### Inclusion Criteria

• 18 years of age or older
• Patients attending Sagarmatha Choudhary Eye Hospital outpatient clinic
• Able to give informed consent and carry out the tests

#### Exclusion Criteria

• Uncorrected distance visual acuity worse than 6/12 in either eye, or any diagnosed macular disease affecting central vision (to reduce patient factors that might affect the repeatability of results)
• Postoperative/intraoperative complications
• Have received mydriatic drops
• Declined to partake in the trial or complete all tests
• Symptoms of COVID-19

### NVA Measurement

**Figure 1.**
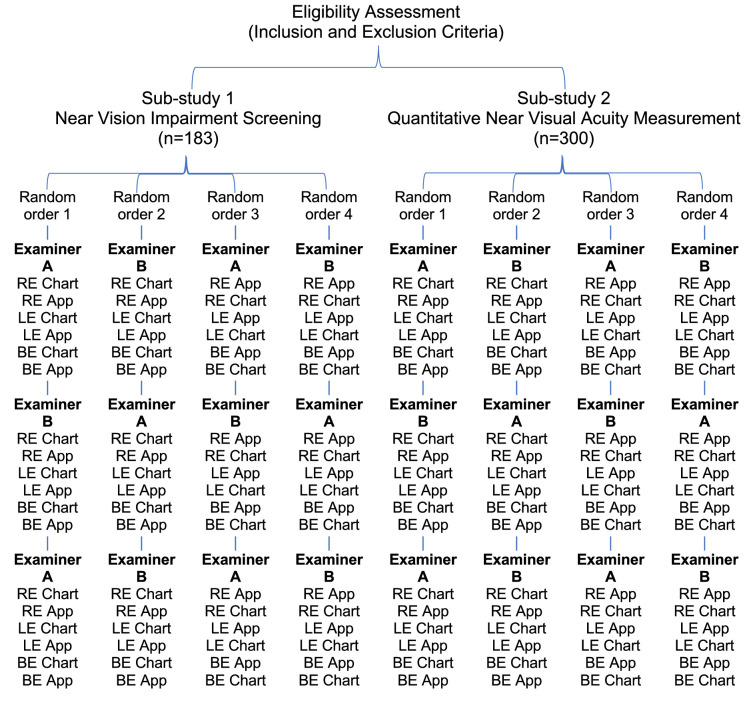
Study flow diagram for the study design. Participants were first assessed for eligibility and then consented and were enrolled in the sub-study in progress at the time (sub-study 1 or 2). They would be automatically allocated to one of four random testing orders, commencing either with examiner A or B and with the chart or app, ultimately undertaking a total of 18 visual acuity tests in the order shown.

Within each sub-study, participants’ uncorrected NVA was tested for monocular right eye vision, monocular left eye vision, and binocular vision, with a conventional Tumbling “E” Near Point Vision Chart and with a PeekNV smartphone-based test using Sony Xperia 10 II smartphone devices (Sony Corporation, Tokyo, Japan). Vision was tested without correction to maximize the range of near acuities available for analysis. This was carried out by two examiners in sequence—first examiner (A or B), second examiner, and again first examiner—thus allowing analysis of inter- and intra-rater repeatability. Therefore, in total, each participant's NVA was measured 18 times (Fig. 1). Data were collected using ODK Collect software, which randomized the order of tests (chart or app first) and the starting examiner. Randomization took place after enrollment of the participant and collection of baseline participant information and was embedded in the app; therefore, it was completely concealed.

The Sony Xperia 10 II was chosen because its pixel density allowed a <5% tolerance in the width of each arm of every selected optotype to be adhered to, in keeping with the European Standards (EN)/International Organization for Standardization (ISO) 8596 standard, with the exception of N10, which had arms 5.6% wider than the theoretical width required for a test at 40 cm.

Testing was performed within 10 × 10 × 8-foot booths constructed from polyvinyl chloride canvas. Within each station, a chin rest was used to stabilize the participant's head position, and a stand angled at 45° was positioned 40 cm away from the participant's eyes. This stand was used to hold the smartphone and Tumbling “E” Near Point Vision Chart (see Supplementary Fig. S1). Rooms were lit by light-emitting diode bulbs and checked every day with a manual lux meter (Restriction of Hazardous Substances [RoHS]-compliant, 2019) to ensure that the ambient light fell within the range of 150 to 180 lux. Illumination was also recorded automatically by a smartphone lux meter. If needed, some adjustments (table position shifting in optimal direction) were made to maintain the symmetry of lighting between two booths. The time taken by each test (from display of first “E” optotype to end of test) was recorded for 132 individual quantitative NVA tests (66 chart and 66 app), and 180 NVI screening tests (90 chart and 90 app).

### Data Collection and Management

Simple demographics, distance visual acuity, and the main eye condition leading to clinic attendance were inputted into a custom-built form using the ODK software application; participants were then guided to station A or B according to the randomization order, and the NV tests were conducted and recorded in the ODK form. The PeekNV test application was integrated into the ODK form, such that test results were automatically recorded. Results of the chart NV test were manually inputted by the examiners. Data collection with this test can be undertaken while offline, allowing for use in remote locations; data synchronization is then carried out later when an internet connection is available. Data were uploaded and transferred via a secure server supported by the London School of Hygiene and Tropical Medicine (LSHTM). No personal identifiers or confidential information were recorded, allowing the transfer of information internationally without risk to data protection. Data were recorded digitally within password-protected devices, and paper records (including consent forms) were stored in locked cabinets. At the end of the project, records will be stored for a minimum period of 7 years, as per legal mandate.

### Data Analysis

All VA measurements were converted to logMAR for analysis. Data cleaning was carried out using Microsoft Excel (Microsoft Corporation, Redmond, WA), and statistical analysis was undertaken using the Stata 16 software package (StataCorp LLC, College Station, TX). Bland–Altman plots were created using R 4.2.0 (National Institutes of Health, Bethesda, MD). The Bland–Altman LoA technique was used to evaluate the test–retest repeatability of quantitative NVA by the PeekNV test and the conventional Tumbling “E” test and to assess agreement between the PeekNV NVA testing with conventional Tumbling “E” testing.

Treating the conventional Tumbling “E” test as a reference standard, the validity of the screening function of PeekNV test for identification of NVI was assessed using Cohen's kappa coefficient, sensitivity, and specificity. The repeatability of screening results by PeekNV and chart was reported by crude agreement percentage and Cohen's kappa, accompanied by 95% confidence intervals (CIs). As Bland–Altman assessment assumes the independence of data, sensitivity, specificity, and kappa agreements for the right eye, left eye, and both eyes for each test were reported separately. A logistic regression model within a generalized estimating equation (GEE) was used to assess the association of demographic characteristics and test elements with the agreement between PeekNV and the conventional test. The difference in time taken by the conventional test and PeekNV test was compared using a linear regression model within the GEE.

### Ethical Approval

The study was approved by the ethics committees of the London School of Hygiene & Tropical Medicine (ref. 22945) and the Nepal Health Research Council (proposal ID 207-2021). Informed consent was obtained from all participants after they were given an explanation of the nature and objectives of the study and examination process. All participants gave written (or thumbprint) consent to participate. The study was conducted in accordance with the tenets of the Declaration of Helsinki.

## Results

### Participant Characteristics

Of the 483 individuals who participated in this validity study, 183 participated in sub-study 1 for NVI screening and 300 participated in sub-study 2 for quantitative NVA measurement (Fig. 1). The participants’ demographics, distance visual acuity, and main eye conditions for the two sub-studies are presented separately in Table 2.

**Table 2.** Characteristics of Study Participants

	Sub-Study 1		Sub-Study 2	
Characteristic	*n*	%	*n*	%
Age groups				
18–39 y	104	56.83	140	46.67
40–49 y	59	32.24	106	35.33
≥50 y	20	10.93	54	18
Gender				
Male	87	47.54	161	53.67
Female	96	52.46	139	46.33
Distance visual acuity				
6/6	90	49.18	148	49.33
6/9	72	39.34	122	40.67
6/12	21	11.48	30	10
Own reading glasses				
No	150	81.97	215	71.67
Yes	33	18.03	85	28.33
Wearing reading glasses				
No	151	82.51	219	73
Yes	32	17.49	81	27
Underlying eye diseases				
Cataract, treated	—	—	1	0.33
Glaucoma	1	0.55	1	0.33
Globe abnormalities	3	1.64	—	—
Minor eye conditions^a^	84	45.9	140	46.67
Posterior segment	1	0.55	—	—
Refractive errors	43	23.5	71	23.67
None	51	27.87	87	29
Total number	183	100	300	100

Sub-study 1 compared PeekNV to chart testing as a screening tool for NVI; sub-study 2 compared PeekNV to chart testing as a quantitative measure of NVA.

a Pterygium, pingueculitis, corneal scar, symptoms of dry eye, blepharitis, lid-related issue, etc.

### Sub-Study 1: Binary NVI Screening

The first sub-study assessed the PeekNV test as a screening tool for NVI according to the WHO definition: inability to read N6 at 40 cm. The prevalence of NVI in the participants’ right eyes was 42.1% (*n* = 77) as measured by the Tumbling “E” chart. The agreement and screening attributes of PeekNV results compared to the Tumbling “E” chart (reference standard) are presented in Table 3. There was 92.9% agreement between the standard chart and app screening results, with a kappa agreement of 0.85. When treating the Tumbling “E” Near Point Vision Chart as the reference standard, PeekNV had a sensitivity of 89.6% and specificity of 95.3% for identifying NVI. The positive predictive value was 93.2% and negative predictive value was 92.7%. Table 4 shows the repeatability of each NV test when it was performed by the same examiner on the same eye of the same person. All comparisons have an acceptable and comparable kappa score for both the PeekNV test and Tumbling “E” chart.

**Table 3.** Accuracy of PeekNV as a NVI Screening Test

Parameter	Point Estimate	Lower Bound 95% CI	Upper Bound 95% CI
Sensitivity	89.61%	85.19%	94.03%
Specificity	95.28%	92.21%	98.35%
Positive predictive value	93.24%	89.61%	96.88%
Negative predictive value	92.66%	88.88%	96.44%
Diagnostic accuracy	92.90%	88.23%	95.80%
Cohen's kappa (unweighted)	0.85	0.708	0.998

NVI defined as vision worse than N6 at 40 cm. The reference test was the conventional Tumbling “E” chart. These results are for the right-eye-first examination only, as when used in other contexts the test will usually only be used once, without practice effect.

**Table 4.** Repeatability of Peek and Chart NVI Screening Tests

	Crude Agreement	Cohen's Kappa Score	Lower Bound 95% CI	Upper Bound 95% CI
App, right eye	90.16%	0.79	0.78	0.80
Chart, right eye	92.35%	0.84	0.83	0.85
App, left eye	90.71%	0.80	0.79	0.81
Chart, left eye	88.52%	0.76	0.75	0.77
App, binocular	90.71%	0.73	0.72	0.74
Chart, binocular	89.07%	0.72	0.70	0.73

The table summarizes the repeatability of the two different tests when used by the same examiner on the same eye of the same person. App refers to the PeekNV smartphone-based digital application; chart refers to the Tumbling “E” Near Point Vision Chart.

Based on a GEE regression model, other factors were found to have no significant impact on the agreement level: level of light or lux (*P* = 0.85); order of tests (i.e., app first or chart first) (*P* = 0.73); examiner (*P* = 0.14); participants’ age (*P* = 0.15); participants’ gender (*P* = 0.19); or the examined eye (i.e., right, left, or binocular) (*P* = 0.86).

### Sub-Study 2: Quantitative NVA Measurement

**Figure 2.**
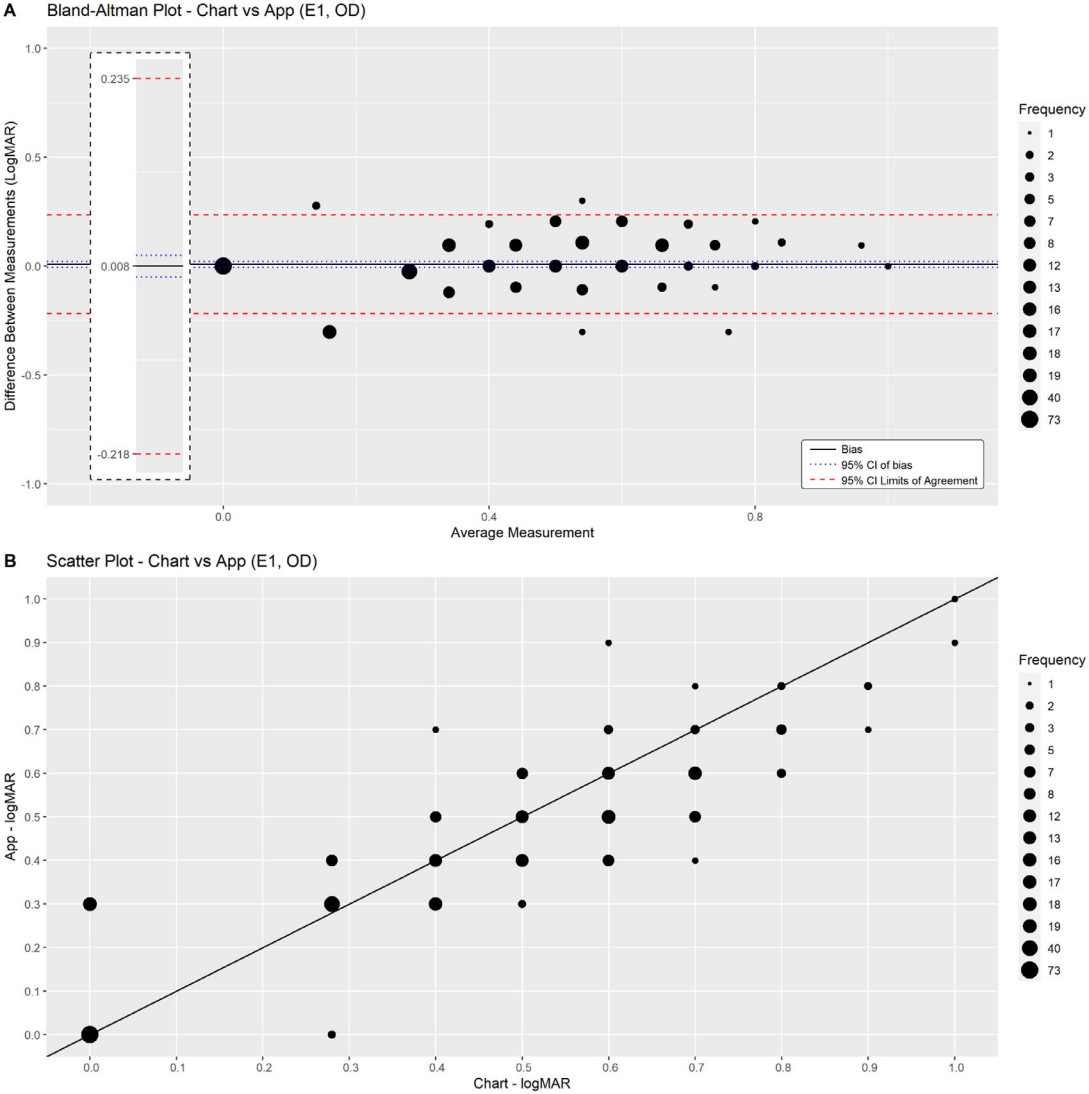
Bland–Altman and scatterplots comparing PeekNV versus chart NVA results (right eye) and the quantitative NVA results from PeekNV smartphone-based testing and the conventional Tumbling “E” Near Point Vision Chart. (A) Bland–Altman plot demonstrating the difference between measurements from the two different testing modalities. The mean difference (bias) between tests is indicated by the solid line, 95% CIs of this difference are indicated by *dotted blue lines*, and 95% LoA are indicated by *dotted red lines*. E1 represents results from first examination; OD indicates results from right eye testing. Results from other tests (left eye and binocular testing) are available in the Supplementary Figures. (B) Scatter plot to represent correlation between testing modalities. Y axis = PeekNV smartphone-based app test results. X axis = Tumbling “E” Near Point Vision Chart test results.

In assessment of right eye data from the first examination, the mean difference (bias) between the PeekNV measurement and Tumbling “E” Near Point Vision Chart measurement was 0.008 logMAR (95% confidence interval [CI], −0.005 to 0.021), and the 95% LoA were within 0.235 to −0.218 logMAR. Correlation (scatterplots) and Bland–Altman plots for comparisons are shown in Figure 2. The LoA and mean difference (bias) in other combinations of paired app and chart near vision examinations (including left eye and binocular, in the first, second and third examinations) are provided in Table 5 and in the Supplementary Figures. As shown, the mean difference in all comparisons is close to 0, with a 95% CI that covers 0. The LoA are also in an acceptable range (close to 0.2 logMar) in all comparisons, meaning that the difference between the app and chart results is not statistically or clinically significant.

**Table 5.** Pairwise Comparisons of Quantitative NVA Results Measured by the PeekNV App and the Conventional Tumbling “E” Chart

Reliability/Repeatability	Examined Eye (*n* = 300)	Mean Difference	95% CI of Difference	95% LoA	Pearson Correlation Coefficient	95% CI of Correlation
App vs. chart	Right eye, E1	0.008	−0.005 to 0.022	−0.218 to 0.235	0.908	0.886 to 0.926
App vs. chart	Left eye, E1	−0.006	−0.019 to 0.007	−0.229 to 0.218	0.908	0.886 to 0.926
App vs. chart	Binocular, E1	−0.016	−0.027 to −0.004	−0.211 to 0.179	0.894	0.869 to 0.915
App vs. chart	Right eye, E2	−0.004	−0.016 to 0.008	−0.208 to 0.2	0.922	0.903 to 0.937
App vs. chart	Left eye, E2	−0.008	−0.02 to 0.005	−0.223 to 0.207	0.907	0.885 to 0.925
App vs. chart	Binocular, E2	−0.007	−0.017 to 0.004	−0.185 to 0.172	0.906	0.883 to 0.924
App vs. chart	Right eye, E3	−0.0005	−0.013 to 0.012	−0.216 to 0.215	0.908	0.886 to 0.926
App vs. chart	Left eye, E3	−0.004	−0.017 to 0.008	−0.223 to 0.215	0.897	0.8724 to 0.917
App vs. chart	Binocular, E3	−0.011	−0.021 to −0.002	−0.174 to 0.152	0.914	0.893 to 0.931
App vs. app	Right eye	0.034	0.021 to 0.047	−0.194 to 0.262	0.907	0.885 to 0.925
Chart vs. chart	Right eye	0.025	0.013 to 0.037	−0.18 to 0.23	0.899	0.875 to 0.919
App vs. app	Left eye	0.027	0.013 to 0.041	−0.214 to 0.268	0.892	0.866 to 0.913
Chart vs. chart	Left eye	0.028	0.017 to 0.040	−0.162 to 0.219	0.913	0.892 to 0.930
App vs. app	Binocular	0.014	0.003 to 0.025	−0.173 to 0.202	0.903	0.880 to 0.922
Chart vs. chart	Binocular	0.019	0.009 to 0.028	−0.148 to 0.185	0.905	0.880 to 0.922

Examinations 1 and 3 were performed by the same assessor (examiner), and examination 2 was carried out by a different assessor. App refers to the PeekNV smartphone-based digital application; chart refers to the Tumbling “E” Near Point Vision Chart. E1, first exam; E2, second exam; E3, third exam.

**Figure 3.**
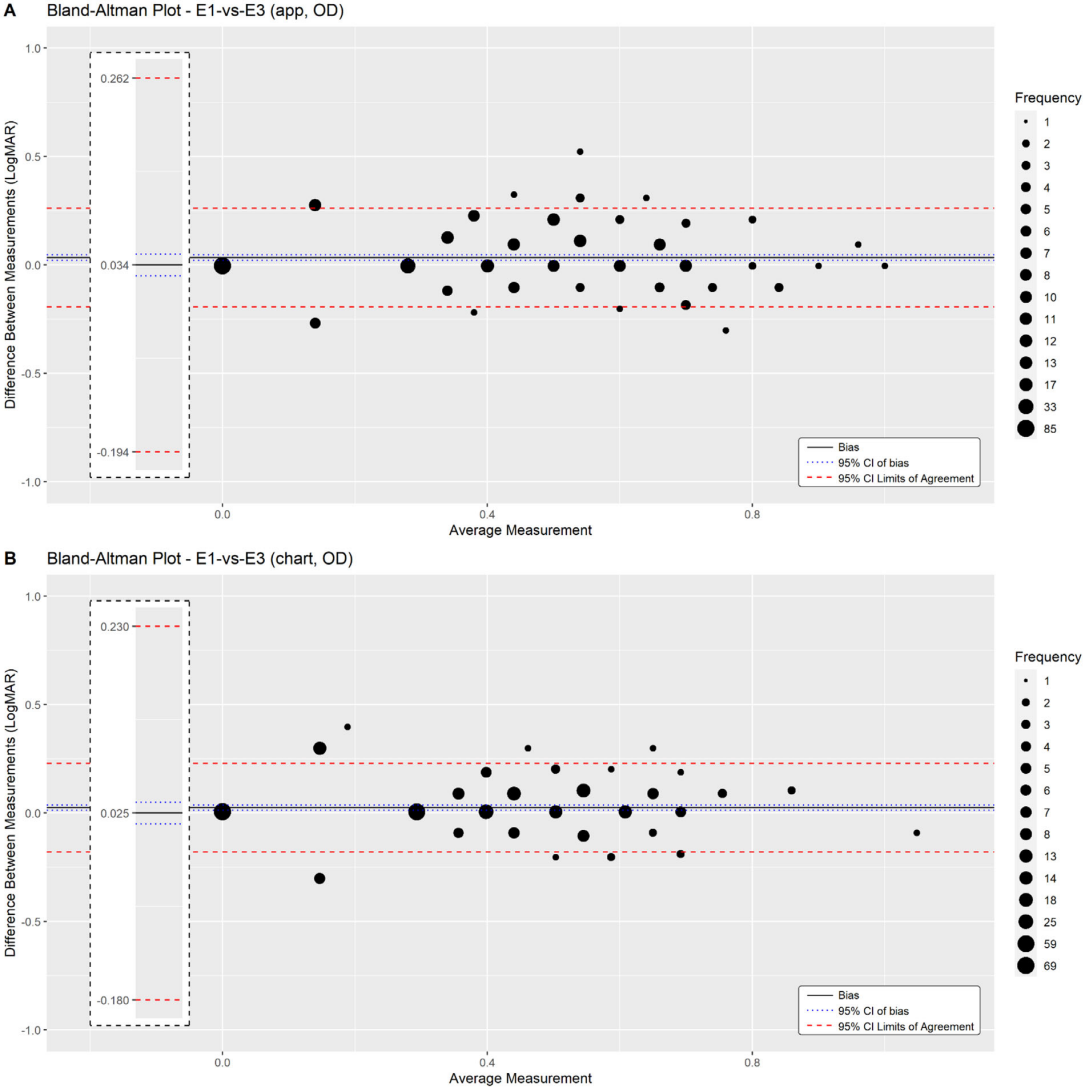
Bland–Altman plots showing the intra-rater repeatability of NVA testing by the PeekNV app and the chart. (A) This Bland–Altman plot compares the right eye NVA results from PeekNV smartphone-based testing during the first examination (E1) and the third examination (E3), both carried out by the same assessor. (B) This Bland–Altman plot compares the right eye NVA results from conventional Tumbling “E” chart testing during the first examination (E1) and the third examination (E3), both carried out by the same assessor. The mean difference (bias) between the test results is indicated by the *solid line*, 95% CIs of this difference are indicated by *dotted blue lines*, and 95% LoA are indicated by *dotted red lines*.

The repeatability of each test was measured by comparison of the same eye tested by the same examiner using the same method. Figure 3A shows the repeatability of the NVA measured by the PeekNV test in the right eye during the first examination. The mean difference between the two PeekNV measurements was 0.034 logMAR (95% CI, 0.021 to 0.047), and the 95% LoA were within −0.194 to 0.262 logMAR. The same two measurements by the conventional Tumbling “E” Near Point Vision Chart (Fig. 3B) had a mean difference of 0.025 logMAR (95% CI, 0.013 to 0.037), and the 95% LoA were within −0.180 to 0.230 logMAR. As shown, the 95% LoA and 95% CI of mean difference of measurements by PeekNV versus PeekNV and chart versus chart are very similar. Similar repeatability of NV scores was found in all other comparisons of eye/exam orders (see Table 4 and Supplementary Figs. S2 to S13).

### Timing of the Tests

**Figure 4.**
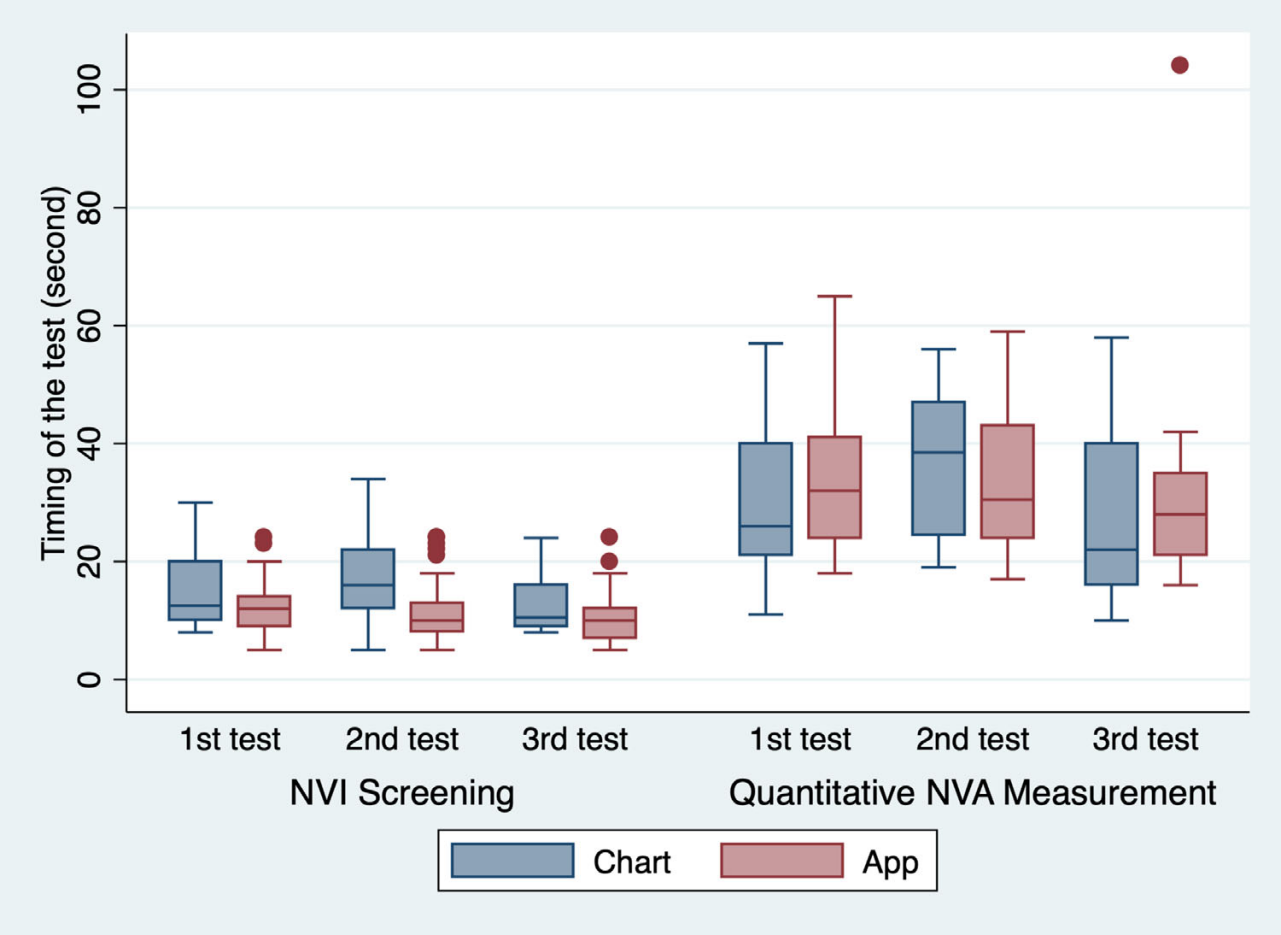
Duration of the near vision tests by the PeekNV app versus the chart. The box plot summarizes the time taken by the PeekNV and chart-based testing. Screening for NVI is shown on the *left* *side* of the graph, and quantitative NVA scoring is shown on the *right side* of the graph. As each participant was tested multiple times, and to allow for practice effects and fatigue, first, second, and third tests (examinations) are shown separately. *Blue boxes* represent chart testing, and *red boxes* represent app testing.

Figure 4 shows the details of test duration in both sub-studies. There was no statistically significant difference between mean time taken to measure NVA with the Tumbling “E” Near Point Vision Chart and PeekNV (31.36 seconds vs. 33.78 seconds). Time taken to identify the presence or absence of NVI with the conventional Tumbling “E” chart was 14.87 seconds (95% CI, 13.49 to 16.24); the mean time with PeekNV was 11.58 seconds (95% CI, 10.52 to 12.64), making PeekNV 3.29 seconds quicker (*P* < 0.001).

## Discussion

Here we report the results of validation studies for a new smartphone-based NVA test, which we present in two formats: (1) a screening test to identify people with NVI (worse than N6), and (2) a quantitative assessment of the degree of NVI. For both forms of the test, we found high levels of agreement with a reference near vision test and high degrees of repeatability in a controlled environment (within and between assessors).

Our results showed that the newly developed PeekNV test identified NVI with a positive predictive value of 93.2%, negative predictive value of 92.7%, sensitivity of 89.6%, and specificity of 95.3%. Agreement of the quantitative NVA results between PeekNV and Tumbling “E” Near Point chart testing was within acceptable limits in 95% of comparisons. Given the dynamic variability of accommodation, some variation in results on retesting is to be expected. Repeatability was similar for the standard chart and PeekNV testing. The kappa agreement was higher for the chart in right-eye examinations but was higher for the app than the chart in left-eye and binocular examinations within our study setting (Table 4). Therefore, we believe the degree of variation in PeekNV results is within acceptable limits. The PeekNV test was quicker than the standard chart when used to screen for NVI, though not clinically significant, and there was comparable timing with no significant difference between mean test time for the quantitative PeekNV NVA measurement and chart-based testing. Overall, the PeekNV test performed well and was no less repeatable than the Tumbling “E” Near Point chart test, and it was comparable in accuracy.

PeekNV was tested and validated against the Tumbling “E” Near Point Vision Chart as a test of both monocular and binocular acuity and impairment, confirming that either option can be used. Although monocular visual acuity is a more helpful indicator of ocular pathology, binocular acuity is more closely correlated with an impact on quality of life, making both valuable metrics.^26^

Many available digital near vision tests either had insufficient evidence of validity or attributes that were not compatible with the needs we found in the expert survey prior to developing the PeekNV app. Important missing attributes included adequate evidence of reliability, appropriateness for individuals with low literacy, and timing of the test. Some of the existing digital tests were designed for individuals to use for at-home testing^27^ and are often too time consuming to be considered for mass screening or survey (e.g., 172 seconds for PDI Check, or much longer for some tests).^28^ Some validated tests were unfortunately no longer available, such as MAVERIC, or were only available in limited regions.^29^^–^^31^

Our study has a number of limitations. First, as agreement was found to be superior in those without NVI, agreement between the tests might be lower in a population of exclusively presbyopic individuals. One explanation for this finding might be accommodation fatigue in those who are struggling to see the smaller optotypes. Our study cohort included a large proportion of younger, pre-presbyopic adults, and further research could include a predominantly older participant group. Of course, accuracy in both those with and without presbyopia is important for a useful screening test, and our cohort included participants with a wide range of NVAs. Considering a key purpose of our test is extensive population-based screening, we believe this test can be used with high confidence. Second, in order to directly compare PeekNV with a chart and minimize confounders, this study was carried out in a standardized, controlled setting. Visual acuity results (with any testing tool) are likely to vary much more if, for example, testing distance is not so rigorously controlled. Third, the Tumbling “E” Near Point Vision Chart was treated as a reference standard, as it is a widely accepted NVA testing device. However, unlike in distance acuity measurement, there is a lack of consensus on a reference standard test for NVA. Suggestions have been made on optimal chart choice (e.g., for children of different ages), but in practice various “conventional” charts are used, according to the personal preference of the assessor.^32^ This lack of a widely agreed-upon standardized near acuity test makes measuring and tackling NVI more challenging and may limit interpretation of the results. Finally, this validity study focused only on adult participants. Although functional NVA testing could be carried out in children without cycloplegia, cycloplegic testing would be required for identification of hyperopia, due to their stronger accommodative abilities.

Further research involving this test could involve validation using other devices (which must meet pixel density specifications) and in other populations and settings, including less standardized testing environments. In addition, results produced during its use in various research projects and screening programs will be collected and analyzed. There is also potential for the development of additional test types, such as continuous text reading tests.

Globally, untreated NVI is estimated to affect >500 million individuals.^9^ This number is expected to increase due to growing aging populations and an increased burden of presbyopia. eREC acts as a key indicator of eye care service uptake and achievement of universal health coverage.^10^ Calculation of near vision eREC requires measurement of whether individuals have uncorrected NVA worse than N6 at 40 cm, but whose presenting NVA is N6 or better, due to refractive correction (met need). This requires a tool for measurement of NVA to be used as part of a population-based survey. Conventional charts can be costly and may become damaged, and access can be a challenge in low-income settings. A rapid and reliable test that could be integrated into different digital platforms such as the RAAB could serve this purpose, as well as the potential to develop a rapid presbyopia-specific population survey tool.^33^^,^^34^ Digital distance visual acuity testing has proved popular in community/school screening programs and research, in part due to increased screening quality and the improved ease of data management and automatic recording it enables.^20^ This in turn can improve service uptake.^35^ Combining this with digital NVA testing, particularly in populations at risk of presbyopia who can be identified, diagnosed, and treated in the same visit, will facilitate the identification and management of NVI. It may also make NVA measurement more appealing to organizers of community health surveys and surveillance who are already using digital devices for data collection. The PeekNV test we have validated in this study could serve this purpose.

## Supplementary Material

Supplement 1

Supplement 2

Supplement 3

Supplement 4

Supplement 5

Supplement 6

Supplement 7

Supplement 8

Supplement 9

Supplement 10

Supplement 11

Supplement 12

Supplement 13

Supplement 14
