# Supplementary material for: Development and Validation of a Digital (Peek) Near Visual Acuity Test for Clinical Practice, Community-Based Survey, and Research
Source: Transl Vis Sci Technol. 2022 Dec 30;11(12):18. doi: 10.1167/tvst.11.12.18 (PMC9807182; doi:10.1167/tvst.11.12.18)
Supplement: Supplement 1 [file tvst-11-12-18_s001.pdf]

Supplementary Figures

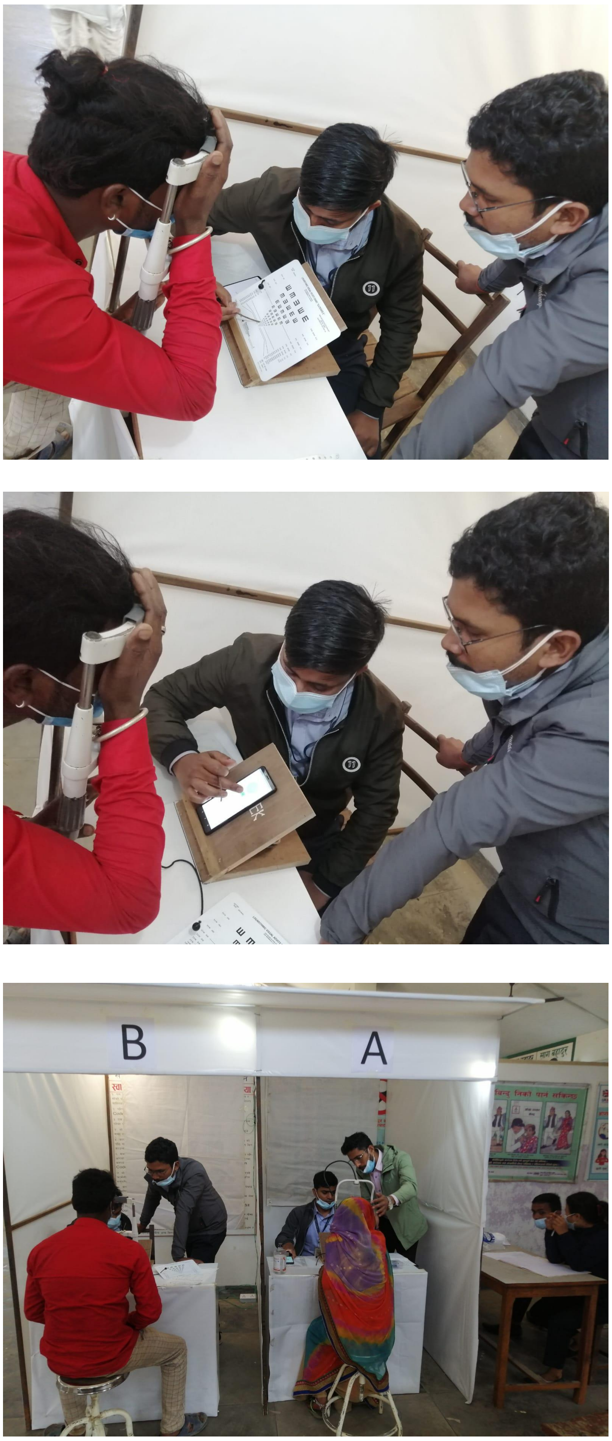

Supplementary Figure 1 (A-C): Validity Study Set-Up. A: Positioning of participant with Tumbling "E" Near Point Vision Chart. B: Positioning of participant with smartphone PeekNV testing. C: Testing booths.
