# Supplementary material for: Development and Validation of a Digital (Peek) Near Visual Acuity Test for Clinical Practice, Community-Based Survey, and Research
Source: Transl Vis Sci Technol. 2022 Dec 30;11(12):18. doi: 10.1167/tvst.11.12.18 (PMC9807182; doi:10.1167/tvst.11.12.18)
Supplement: Supplement 5 [file tvst-11-12-18_s005.pdf]

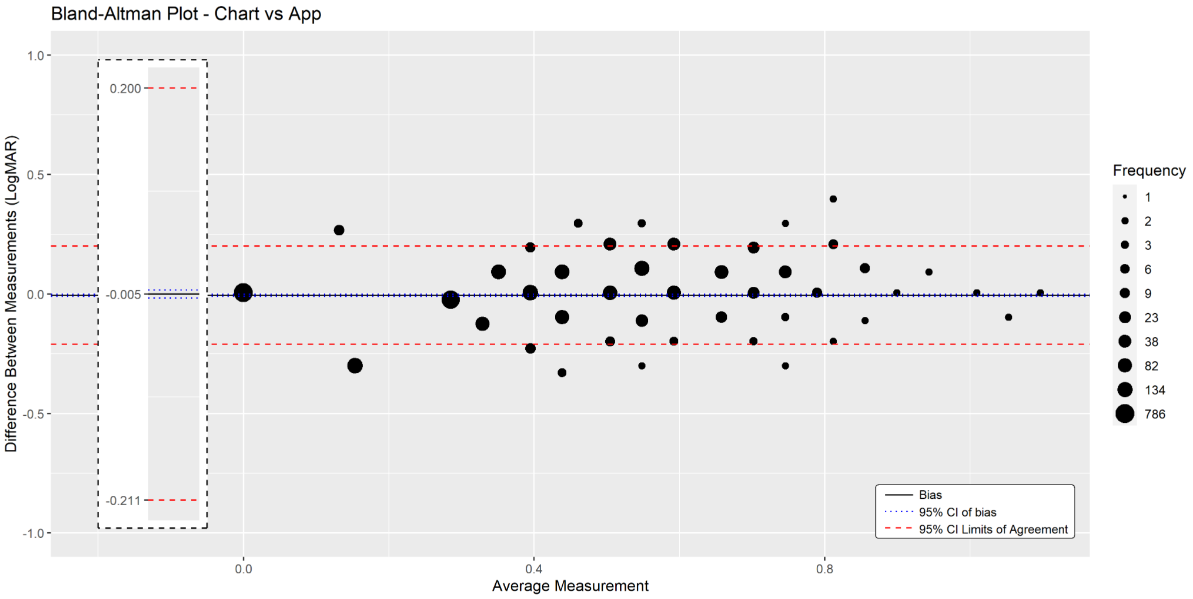

Supplementary Figure 5: Bland-Altman plot – Comparison of PeekNV vs chart Near Visual Acuity results, all eyes and examinations.
