# Supplementary material for: Development and Validation of a Digital (Peek) Near Visual Acuity Test for Clinical Practice, Community-Based Survey, and Research
Source: Transl Vis Sci Technol. 2022 Dec 30;11(12):18. doi: 10.1167/tvst.11.12.18 (PMC9807182; doi:10.1167/tvst.11.12.18)
Supplement: Supplement 7 [file tvst-11-12-18_s007.pdf]

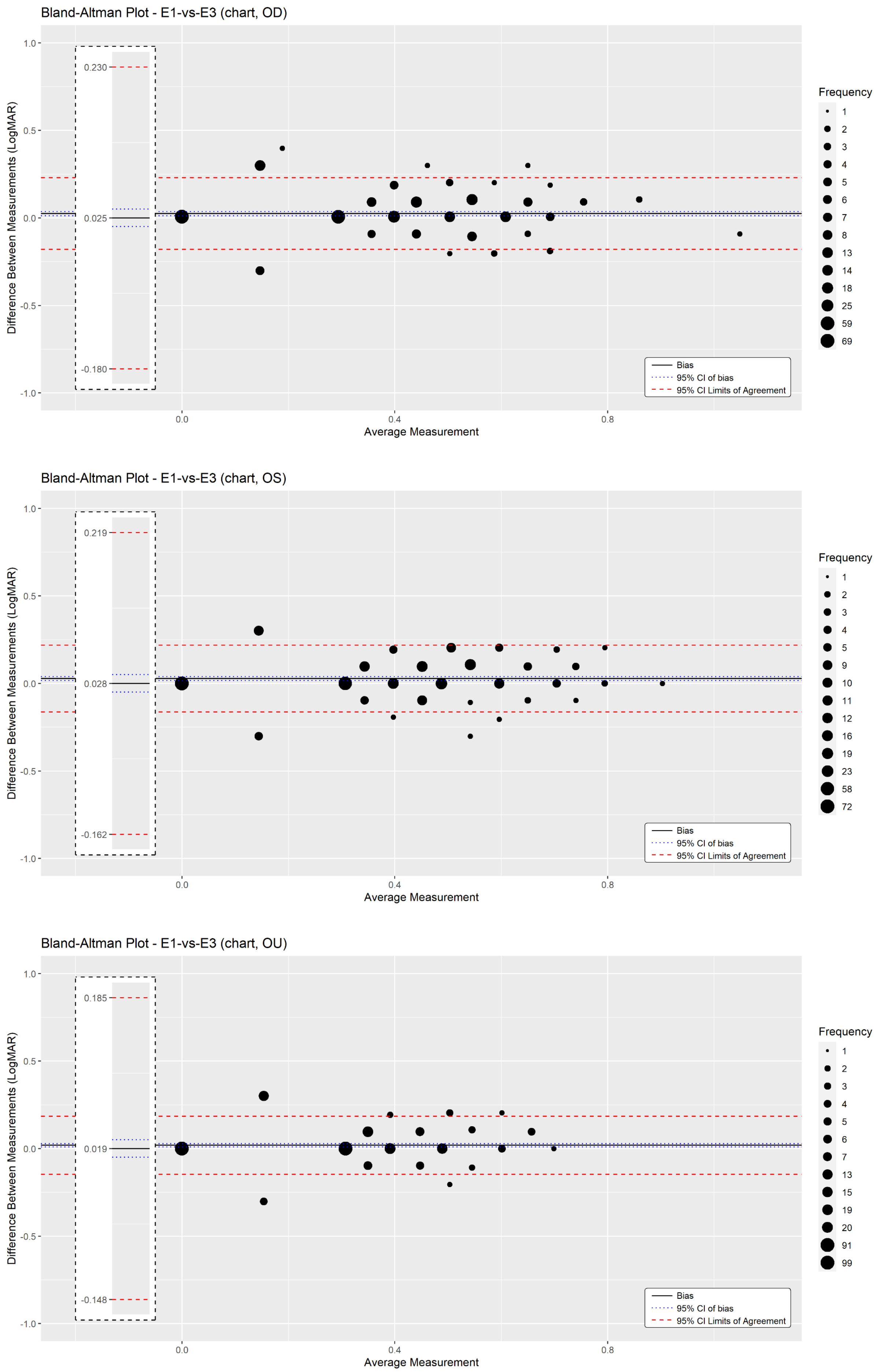

Supplementary Figure 7 (A-C): Bland-Altman plots – Intra-tester test-retest repeatability of chart Near Visual Acuity results. A: OD, right eye. B: OS, left eye. C: OU, both eyes. E1: First examination. E3: Third examination.
