# Supplementary material for: Development and Validation of a Digital (Peek) Near Visual Acuity Test for Clinical Practice, Community-Based Survey, and Research
Source: Transl Vis Sci Technol. 2022 Dec 30;11(12):18. doi: 10.1167/tvst.11.12.18 (PMC9807182; doi:10.1167/tvst.11.12.18)
Supplement: Supplement 10 [file tvst-11-12-18_s010.pdf]

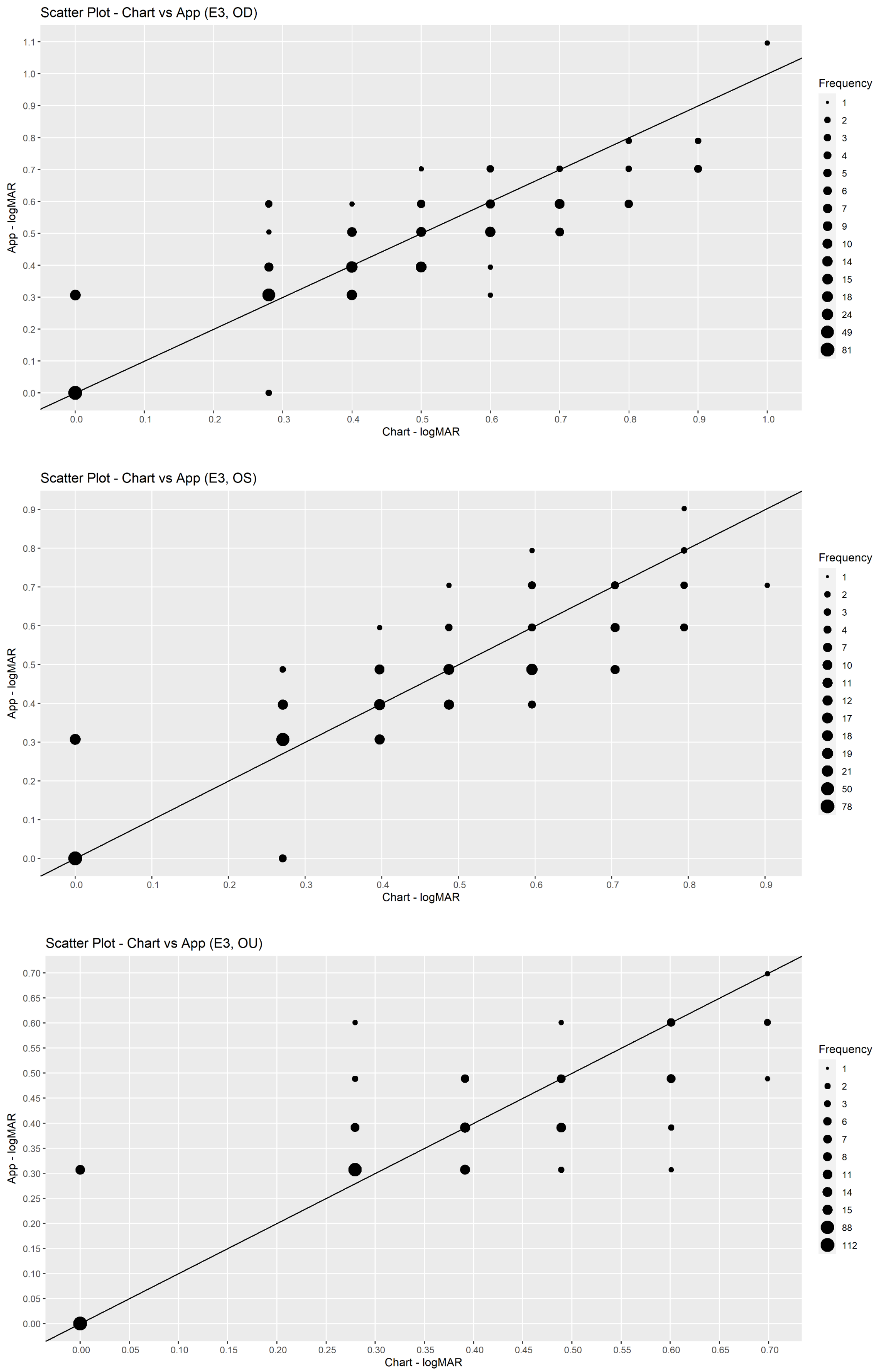

Supplementary Figure 10 (A-C): Scatter plots – Comparison of PeekNV vs chart Near Visual Acuity results, third examination. A: OD, right eye. B: OS, left eye. C: OU, both eyes. E3: Third examination.
