# Supplementary material for: Development and Validation of a Digital (Peek) Near Visual Acuity Test for Clinical Practice, Community-Based Survey, and Research
Source: Transl Vis Sci Technol. 2022 Dec 30;11(12):18. doi: 10.1167/tvst.11.12.18 (PMC9807182; doi:10.1167/tvst.11.12.18)
Supplement: Supplement 14 [file tvst-11-12-18_s014.pdf]

Sub-study 1

To have a sample size in sub-study 1 we consider to calculate the kappa statistics based on the following formula provided by Fleiss:

$$n = Z\alpha^2/\delta^2 \times Po(1-Po)/(1-Pe)^2$$

α =Determine the confidence level = 5%

Po = The prevalence for fail = 30%

δ = Maximum tolerable error = 20%

Pe = Expected agreement by chance (calculated from Po) = 58%

n = Required sample size = 115

Sub-study 2

We have calculated the sample size based on Bland-Altman Limits of Agreement as follows:

$$n=(2+z1-\Upsilon/2^2)[tinv(1-\beta/2,n-1,t1-\alpha/2,n-1)]^2SD^2 / 2(z1-\Upsilon/2SD-\delta)^2$$

This could be obtained only with an iterative method as the tinv used in this formula is related to sample size (n). In fact, this parameter is based on the final sample size and was calculated by software (Medcal (version 19.6.0)) using following elements:

α = Type I error = 5%

1-β = Power = 80%

Υ = The percentage of LoA = 95%

SD = The expected standard deviation of difference = 0.087

δ = Maximum tolerable difference = 0.2

Tinv = The inverse of non-central t distribution = Obtained from iterative calculation

N = Required sample size = 273

Supplementary Information: Sample Size Calculations
